# Protective Efficacy of Novel Oral Biofilm Vaccines against *Photobacterium damselae* subsp. *damselae* Infection in Giant Grouper, *Epinephelus lanceolatus*

**DOI:** 10.3390/vaccines10020207

**Published:** 2022-01-28

**Authors:** Feng-Jie Su, Meei-Mei Chen

**Affiliations:** Department of Veterinary Medicine, National Taiwan University, No. 1, Section 4, Roosevelt Road, Taipei 10617, Taiwan; d06629003@ntu.edu.tw

**Keywords:** *Photobacterium damselae* subsp. *damselae*, oral biofilm vaccine, *Epinephelus lanceolatus*

## Abstract

Photobacterium *damselae* subsp. *damselae* is a pathogen that mainly infects a variety of fish species. There are many antibiotic-resistant strains of *Photobacterium damselae* subsp. *damselae*. In a previously published article, we described the production method for a novel oral biofilm vaccine. In the study reported herein, we confirmed the protective effect of the oral biofilm vaccine against *Photobacterium damselae* subsp. *damselae*. Twenty-eight days after vaccination, phagocytosis increased by 256% relative to the control group. The mean albumin–globulin ratios of the vaccine groups were significantly lower than the mean albumin–globulin ratios of the control group. There were no significant intergroup differences in lysozyme activity. Mean IgM titers were significantly higher in the vaccine group than in the control group. There was a significant upregulation of the TLR 3, IL-1β, and IL-8 genes in the spleen 28 days after vaccination. The cumulative mortality of the control fish was 84% after challenging fish with the *Photobacterium damselae* subsp. *damselae*, while the cumulative mortality of the oral biofilm vaccine (PBV) group was 32%, which was significantly higher than those of the whole-cell vaccine (PWV) and chitosan particle (CP) groups. There is minimal published research on the prevention and treatment of *Photobacterium damselae* subsp. *damselae* infection; therefore, this oral biofilm vaccine may represent a new method to fill this gap.

## 1. Introduction

*Epinephelus lanceolatus* (giant grouper) is taxonomically classified among the Chordata, Neopteridae, Perciidae, and Serranidae in the genus Epinephelus. The species is an important cultured fish species in Asia [1]. In the process of aquaculture, it is highly susceptible to viral and bacterial infections, which results in decreased production.

*Photobacterium damselae* subsp. *damselae* is a Gram-negative bacterium that mainly infects a variety of marine fish. Clinical symptoms include hemorrhage of the liver, spleen, and kidneys [2,3]. *Photobacterium damselae* subsp. *damselae* was first named *Vibrio damsel* until it was renamed in 1995 after 16S rRNA gene sequence analysis. *Photobacterium damselae* has two main subspecies, *Photobacterium damselae* subsp. *damselae* and *Photobacterium damselae* subsp. *piscicida*, both of which are associated with high rates of mortality among marine species [4,5,6,7].

There are many antibiotic-resistant strains of *Photobacterium damselae* subsp. *damselae* [8]. Therefore, vaccination is an important strategy in reducing infectious diseases among fish and other marine animals. The vaccine development process must closely consider standards related to safety, cost, and efficacy. The most commonly used vaccination methods are injection, immersion, and oral administration [9]. Oral vaccines are easy to administer to fish of different sizes and can effectively deliver antigens to the gut-associated lymphoid tissue (GALT) [10]. Multiple-immunogenic biofilm vaccines have demonstrated localization and distribution of antigens in the gut and lymphoid tissues in large amounts for long periods following oral vaccination [11]. When oral vaccines are delivered via the gut, local and systemic immune responses will be elicited, which are reflected in high amounts of circulating immunoglobulin M (IgM) [12,13,14]. Oral biofilm vaccines have been confirmed to have protective effects for many fish, such as *Clarias batrachus* [15], *Lates calcarifer* [16], *Labeo rohita* [17], *Oreochromis* sp. [18], *Cyprinus carpio* [11,19], and *Mugil cephalus* [20].

In this study, using previously demonstrated methods [20], we cultivated *Photobacterium damselae* subsp. *damselae* biofilm formation on chitosan particles with an inactivation step involving formalin. We took oral biofilm vaccines, whole-cell vaccines, and chitosan particles and confirmed the protective effects of the different vaccines by monitoring fish survival after an experimental challenge.

## 2. Materials and Methods

### 2.1. Ethical Considerations

The Centre for Research Animal Care approved this animal experimental study, which had oversight from the Animal Care Use Committee of the National Taiwan University (protocol no. B201900064).

### 2.2. Fish Maintenance

We purchased giant groupers from a giant grouper farm in Kaohsiung, Taiwan. In total, 136 giant groupers were bred in a 500 L fiber-reinforced plastic (FRP) tank in sea water (35 ppt) at 26 °C. We reared the fish (10 g ± 0.7 g) in an indoor circulating water system at the Department of Veterinary Hospital, National Taiwan University (Taipei, Taiwan) at a regulated temperature of 26 ± 2.0 °C. To confirm that the fish were free from *Photobacterium damselae* subsp. *damselae* infection before conducting the experiments, we harvested the spleens of five randomly selected fish for bacterial isolation and polymerase chain reaction (PCR) analysis [21].

#### 2.2.1. Bacterial Strains

We isolated *Photobacterium damselae* subsp. *damselae* from fish farm outbreaks among moribund giant groupers in Taiwan. Blood agar (Oxoid^TW^, Creative Media Plate, New Taipei City, Taiwan) was used to isolate *Photobacterium damselae* subsp. *damselae* colonies, which were identified by sequencing 16S rRNA genes [21]. Individual colonies were cultured in in brain–heart infusion broth (BHI; HiMedia, Creative Media Plate, New Taipei City, Taiwan) containing 1% NaCl at 28 °C.

#### 2.2.2. Cultured and Quantified *Photobacterium damselae* subsp. *damselae* Biofilm

The Dimethylmethylene Blue Assay (DMMB) method was used for biofilm quantification, followed by data conversion into colony-forming units. We cultured *Photobacterium damselae* subsp. *damselae* at a density of 10^3^ CFU/mL in 100 mL of BHI with 1% NaCl and 10 mg/mL of chitosan particles and rotated the preparation at 100 rpm. We calculated CFU/mL values following a method from a previous study [20]. The DMMB method was used to quantitate the biofilm [22]. Using a spectrophotometer (BioPhotometer, Eppendorf, Taiwan), we measured *Photobacterium damselae* subsp. *damselae* biofilm samples at 620 nm and then used scanning electron microscopy (SEM) to ensure that the chitosan particles were encapsulated in the biofilm. We used a JEOL JSM-7800F (JEOL Ltd., Tokyo, Japan) scanning electron microscope at 15 kV to view and photograph the SEM samples, with a 15–17 mm working distance.

#### 2.2.3. Preparation of Feed-Based *Photobacterium damselae* subsp. *damselae* Biofilm Vaccine (PBV), *Photobacterium damselae* subsp. *damselae* Whole-Cell Vaccine (PWV), and Chitosan Particle (CP)

We cultured *Photobacterium damselae* subsp. *damselae* bacteria in 100 mL of BHI broth containing 1% NaCl and 10 mg/mL chitosan particles and spun the culture mixture at 100 rpm and 28 °C for 48 h. We then harvested the biofilms by centrifugation at 300 rpm for 10 min at 4 °C and washed them three times with sterile PBS. We collected 1 mL to homogenize the biofilm and serial dilution incubated the plates at 28 °C for 24 h, followed by calculation of CFU/mL values. Then, 2% (*w*/*v*) formalin was used to inactivate the final biofilm over 24 h. Sterile PBS was used to wash the inactivated biofilm three times. We stored the biofilm vaccine at 4 °C. To prepare the whole-cell vaccine, we inoculated the described strain in BHI containing 1% NaCl and rotated the preparation at 100 rpm at 28 °C for 7 h. Pellet harvesting was achieved by centrifugation at 10,000 rpm for 10 min, after which the pellet was washed three times with PBS. We collected 1 mL of whole-cell serial dilution and grew the cells on BHI agar plates at 28 °C for 24 h, followed by calculation of CFU/mL values and cell inactivation with 2% (*w*/*v*) formalin over 24 h. We washed inactivated whole cells three times with sterile PBS and stored the whole-cell vaccine at 4 °C. The chitosan particle preparation method was modified from that used in a previous study [20]. We used a feed-based method modified from a previous study [18,20]. The commercial feed included fish meal (25%), shrimp powder (40%), groundnut oil cake (18%), vegetable oil (5.8%), vitamin A (44,100 IU/kg), vitamin D3 (7910 IU/kg), vitamin C (1200 mg/kg), and vitamin E (112 mg/kg) homogenized into a powder. We then incorporated inactivated 10^10^ cfu/mL biofilm vaccine (PBV), 10^10^ cfu/mL whole-cell vaccine (PWV), or 10 mg chitosan particle (CP) into the feed.

### 2.3. Vaccination

**Figure 1:**
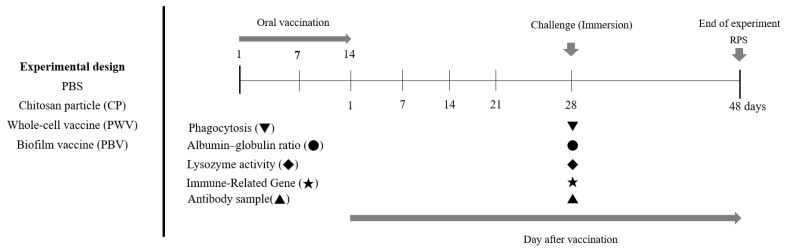
Graphical overview of the vaccination/challenge schedule and sampling time points.

The fish were divided into four groups of 25, and each group was moved to separate 80 L FRP tanks: (1) the PBV group, (2) the PWV group, (3) the CP group, and (4) the PBS group. Vaccination was carried out for 14 consecutive days for each group. We analyzed the collected samples for immune-related genes 28 days after vaccination, at which time we also conducted blood analyses to measure phagocytosis, lysozyme Assay, the albumin–globulin (A/G) ratio, and IgM. Each group was challenged with *Photobacterium damselae* subsp. *damselae* 28 days after vaccination, after which we conducted survival analyses. The timeline of vaccination, sampling, and bacteria challenge is shown in Figure 1.

### 2.4. Phagocytosis Analyses

The collected-leukocytes method was modified from a previous study [23]. We anesthetized each giant grouper with tricaine mesylate (MS-222) in seawater. Using heparinized syringes and vacutainer tubes (BD Microtainer, New Jersey, NJ, USA) containing sodium heparin, we drew 200-µL blood samples from each fish by caudal venipuncture and stored the blood at 4 °C with gentle stirring. Blood samples were pooled in batches of five and diluted at 4 °C with 3 mL normal saline. Next, we collected 3 mL of diluted blood and stored it at 4 °C with gentle stirring until use. The 3 mL diluted blood suspension was added to 4 mL Pancoll (PAN-Biotech, Aidenbach, DE2, Germany) with a density of 1.077 g/mL then centrifuged (800× *g*, 30 min, 4 °C). Leukocytes were collected, washed, resuspended in RPMI-1640 medium, and cultured at 25 °C in 5% CO_2_ for 24 h. After culturing, we collected the adherent cells with 0.25% trypsin EDTA and washed them three times with RPMI-1640 medium followed by resuspension in RPMI-1640 medium. We used the trypan blue exclusion method to determine cell viability, and the suspension was adjusted to an adequate concentration. Analysis of phagocytosis was performed using a pHrodo™ Red *E. coli* BioParticles™ Conjugate for Phagocytosis kit (Invitrogen^TW^, New Taipei City, Taiwan) according to the manufacturer’s instructions. We used the following formula for phagocytosis analysis: phagocytosis = (experimental phagocytosis/PBS control phagocytosis) × 100%.

### 2.5. Albumin–Globulin Ratio Analyses

We calculated A/G ratios in the sera using the method described by Thanga Viji et al. [24]. The serum samples were analyzed for total protein using the Bradford method (Omics bio, New Taipei city, Taiwan). The albumin was analyzed using the bromocresol green method (Formosa Biomedical Technology Corp, Taipei City, Taiwan). The globulin was analyzed by subtracting the albumin value from the total protein value. Finally, the A/G ratio was determined.

### 2.6. Serum Lysozyme Assay

We measured the serum lysozyme activity using a method modified from a previous study [25]. Briefly, we prepared a standard suspension of 0.1 mg/mL of *Micrococcus lysodeikticus* (Sigma-Aldrich, Burlington, MA, USA) in pH 6.0 phosphate buffer (Omics bio, New Taipei City, Taiwan). We then added 50 µL of giant grouper serum to 1 mL of the bacterial suspension, after which the absorbance reduction was recorded at 0.5 and 4.5 min intervals at 450 nm on a spectrophotometer (BioPhotometer, Eppendorf, Hamburg, DE6, Germany). A reduction in absorbance of 0.001/min was considered to be 1 unit of lysozyme activity.

### 2.7. IgM Assay

We collected serum antibodies using a method modified from a previous study [18]. Briefly, we randomly collected four fish from each group and sedated them with 30 ppm Tricaine mesylate (MS-222) in sterile sea water. We drew blood from the caudal vein using a 1 mL disposable syringe (27 G) without anticoagulant, after which serum was extracted by 2500 rpm centrifugation for 15 min. We used sterile sea water to observe the state of the fish, which were returned to the experimental groups after they recovered and resumed swimming. Enzyme-linked immunosorbent assay (ELISA) was used to determine the titers of antibodies against *Photobacterium damselae* subsp. *damselae*. We measured antibodies following a method from a previous study [20].

### 2.8. Immune-Related Genes Expression by qRT-PCR

The fish were euthanized via administration of an anesthetic agent and their abdominal cavities were cut open to obtain their spleens. We extracted total RNA from the fish spleens using an RNA kit (Geneaid Co., Ltd., New Taipei City, Taiwan) according to the manufacturer’s instructions. We synthesized DNA and performed real-time PCR (Applied Biosystems Ltd., Foster City, CA, USA) following a method from a previous study [20]. For real-time PCR, the specific primer pairs were designed as shown in Table 1 [26,27,28]. We used the β-actin gene as a housekeeping gene, and it was amplified using β-actin F and β-actin R gene-specific primers. We ran all samples in triplicate, and each assay was repeated three times. We evaluated relative gene expression levels using the 2^−ΔΔCT^ method.

### 2.9. Challenge with Photobacterium damselae subsp. damselae

We challenged the fish by immersion with 10^7^ CFU/mL *Photobacterium damselae* subsp. *damselae* at 28 days after vaccination. We observed the challenged fish for the next 20 days, and deaths were recorded daily. The relative percentage survival (RPS) was calculated according to the following formula: RPS = (1 − % mortality of immunized group/% mortality of PBS group) × 100%.

### 2.10. Statistical Analysis

We used GraphPad Prism software (Version 8.0; GraphPad Software, Inc., San Diego, CA, USA) to generate graphs and perform statistical analyses. The results are presented as means ± SD of triplicate experiments. We used one-way ANOVA followed by Tukey’s multiple comparison tests to analyze phagocytosis, A/G ratio, antibody response, and relative mRNA expression. We used the log-rank test to compare cumulative survival among the PBV group, the PWV group, the CP group, and the PBS group.

## 3. Results

### 3.1. Scanning Electron Microscopy(SEM) Observation of Photobacterium damselae subsp. damselae Biofilm

**Figure 2:**
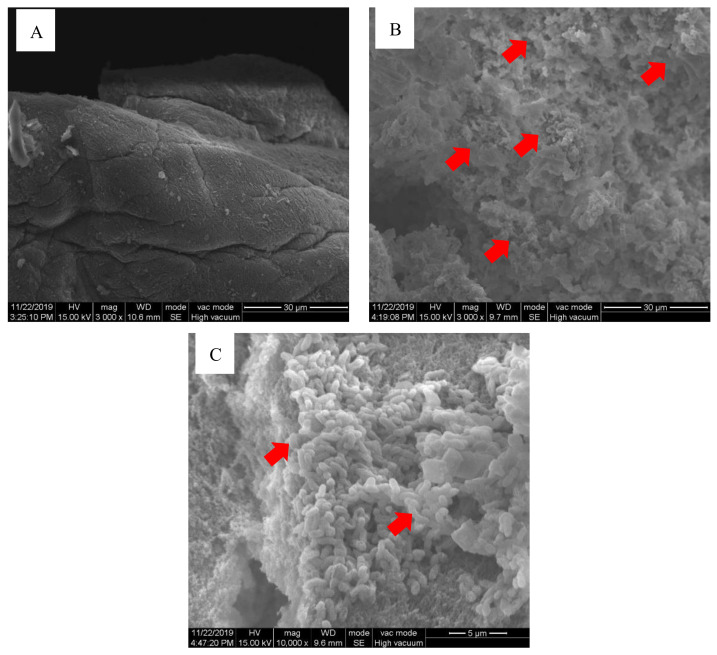
Scanning electron microscopy (SEM) micrograph showing a *Photobacterium damselae* subsp. *damselae* biofilm growth on a chitosan particle. (**A**) Chitosan particle surface without *Photobacterium damselae* subsp. *damselae*. (**B**) Biofilm growth on a chitosan particle at 48 h (arrows: *Photobacterium damselae* subsp. *damselae* biofilm). Magnification: 3000×; bars = 30 μm. (**C**) Biofilm growth on a chitosan particle at 48 h (arrows: *Photobacterium damselae* subsp. *damselae* biofilm). Magnification: 10,000×; bars = 5 μm.

Before the oral administration of PBV was evaluated, *Photobacterium damselae* subsp. *damselae* was grown on chitosan particles to produce a biofilm. Figure 2A shows a chitosan particle (mag. 3000×). Figure 2B shows *Photobacterium damselae* subsp. *damselae* formation on a chitosan particle (mag. 3000×) after 48 h of incubation. Figure 2C shows *Photobacterium damselae* subsp. *damselae* formation on a chitosan particle (mag. 10,000×) after 48 h of incubation.

### 3.2. Innate Immune Response after Vaccination

**Figure 3:**
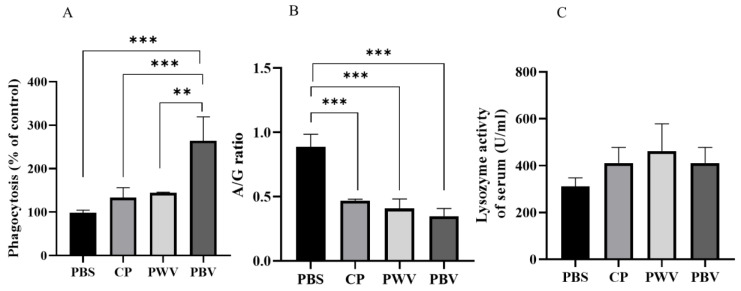
Phagocytosis (**A**), A/G ratio (**B**), and lysozyme activity (**C**) of giant grouper at 28 days post-vaccination. Data are presented as mean ± SD (*n* = 4). *p*-values were calculated by one-way ANOVA (*p* < 0.01 **, *p* < 0.001 ***). PBS: commercial feed with PBS. CP: commercial feed with chitosan particle. PWV: commercial feed with whole-cell vaccine. PBV: commercial feed with biofilm vaccine.

Phagocytosis, lysozyme, and A/G ratio are important defense lines and evaluation indicators of host resistance to bacteria. Phagocytosis of the PBV group was significantly higher than all groups at 28 days after vaccination (Figure 3A). The A/G ratio was significantly (*p* < 0.001) higher in the PBS group than the PBV, PWV, or CP group (Figure 3B). The levels of serum lysozyme were not significantly different among the vaccine groups. In the PBV group, the mean serum lysozyme was 410 U/mL, and in the PWV, CP, and PBS groups, the mean levels were 463, 411, and 311 U/mL, respectively (Figure 3C).

### 3.3. Antibody Production

**Figure 4:**
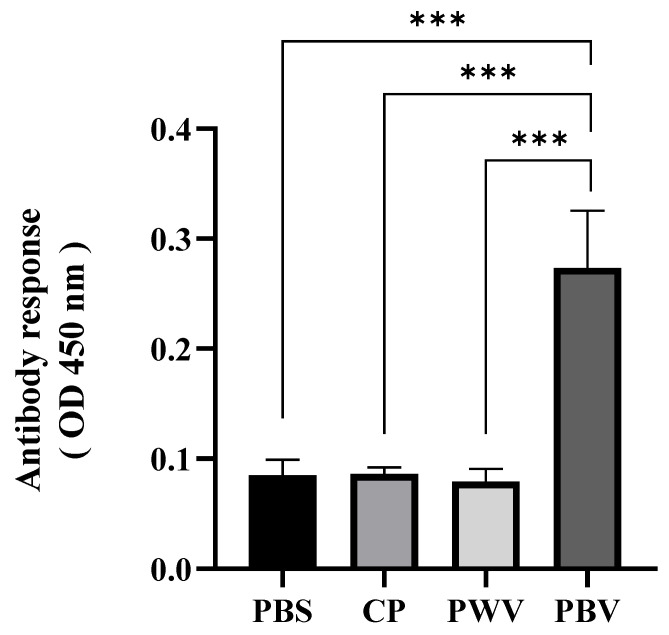
Serum specific antibody detection in vaccinated fish by ELISA. Serum IgM titers of giant grouper at 28 days after vaccination. Data are presented as mean ± standard deviation (*n* = 4). The *p*-values were calculated by one-way ANOVA (*p* < 0.001 ***). PBS: commercial feed with PBS. CP: commercial feed with chitosan particle. PWV: commercial feed with whole-cell vaccine. PBV: commercial feed with biofilm vaccine.

Specific serum antibody (IgM) levels in the different vaccine groups were measured by ELISA and evaluated against *Photobacterium damselae* subsp. *damselae*. In the PBV group, the mean antibody level was significantly higher than those of the other groups (*p* < 0.001) (Figure 4). In the PBV group, the mean OD value was 0.2735, and in the PWV, CP, and PBS groups, the levels were 0.0862, 0.0792, and 0.0853, respectively.

### 3.4. Immune-Related Gene Analysis

**Figure 5:**
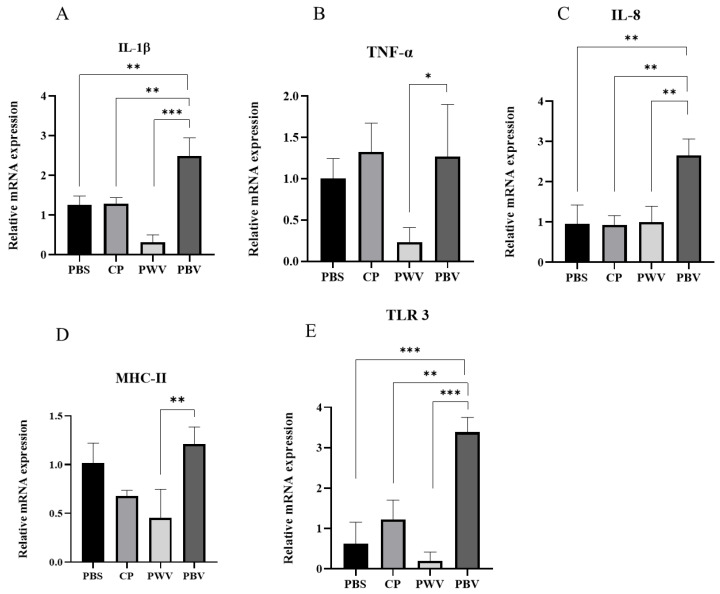
Real-time PCR gene expression levels of (**A**) IL-1β, (**B**) TNF-α, (**C**) IL-8, (**D**) MHC-II, and (**E**) TLR3 in spleen samples at 28 days after vaccination. Data are presented as mean ± standard deviation (*n* = 3), and *p*-values were calculated by one-way ANOVA (*p* < 0.05 *, *p* < 0.01 **, *p* < 0.001 ***). PBS: commercial feed with PBS. CP: commercial feed with chitosan particle. PWV: commercial feed with whole-cell vaccine. PBV: commercial feed with biofilm vaccine.

Expression profiles of immune-related genes were examined in the spleens of fish at 28 days after vaccination. Interleukin 1β (IL-1β), Toll-like receptor 3 (TLR 3), tumor necrosis factor-α (TNF-α), interleukin 8 (IL-8), and major histocompatibility complex class II (MHC-II) were employed to detect the mRNA expression of these genes in the fish spleens (Figure 5). The PBV significantly increased the mRNA levels of IL-1β, IL-8, and TLR 3 compared with the PBS group at 28 days after vaccination. The PBV significantly increased the mRNA levels of TNF-α and MHC-II compared with the PWV group at 28 days after vaccination. In this regard, there were no significant differences among the PWV, CP, and PBS groups at 28 days after vaccination.

### 3.5. Relative Percentage Survival

**Figure 6:**
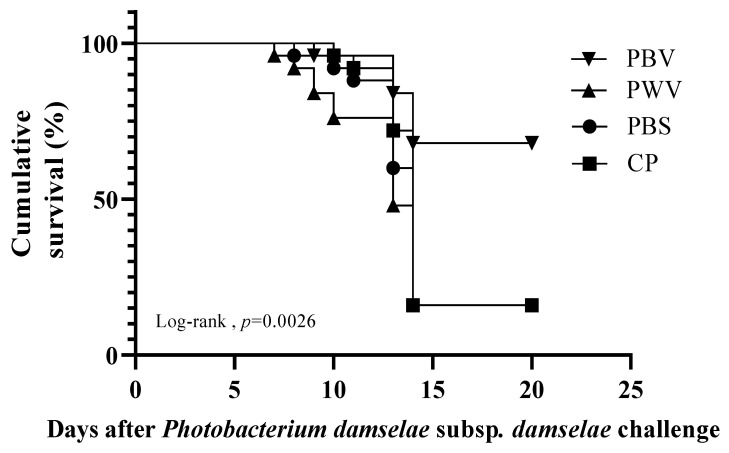
Effect of vaccination on cumulative survival of giant grouper infected with *Photobacterium damselae* subsp. *damselae*. The number of dead fish in each group was counted every day until no deaths were recorded for 5 consecutive days; the total mortality was then calculated. Statistical significance was determined by the log-rank test (*p* < 0.0026). PBS: commercial feed with PBS. CP: commercial feed with chitosan particle. PWV: commercial feed with whole-cell vaccine. PBV: commercial feed with biofilm vaccine.

Finally, we evaluated the survival rate and relative survival rate of the PBV, PWV, and CP groups in the challenge test. The vaccinated fish were challenged with 10^7^ CFU/mL *Photobacterium damselae* subsp. *damselae* at 28 days after vaccination. On the third day after the challenge, we observed that the fish in the PWV and PBS groups had clinical symptoms such as dark body color and loss of balance. The fish death rates increased gradually beginning 8 days after challenge. The survival rates for the groups administered the PBV, PWV, CP, and PBS were 68%, 16%, 16%, and 16%, respectively, at 20 days after vaccination (Figure 6). The relative percent survival (RPS) values were 62, 0, and 0 for the PBV, PWV, and CP groups, respectively (Table 2).

## 4. Discussion

*Photobacterium damselae* subsp. *damselae* causes high mortality in aquaculture fish [2]. Studies have reported that *Photobacterium damselae* forms biofilms and that pathogenic bacteria released from the biofilms may cause disease recurrence [29]. Biofilm formation can cause changes in drug infusion and host responses, including inhibition of inflammation, neutralization of antibodies, and phagocytosis of macrophages [30,31,32]. Biofilm bacteria are encased within a self-produced extracellular matrix (extracellular polymeric substances, or EPS) comprising extracellular DNA (eDNA), proteins, lipids, and exopolysaccharides [33]. Adhesion and hydrolytic activities are essential to the infection and disease symptoms associated with *Photobacterium damselae* subsp. *damselae* [34]. Therefore, we believe that if the biofilm is made into a vaccine, the diversity of antigens could enhance fish immunity and provide a new strategy for vaccine development against infectious diseases [35].

We previously developed a new oral vaccine technology and applied it to *Lactococcus garvieae* with a good protective effect [20]. Therefore, in this study, we applied this method to the production of a *Photobacterium damselae* subsp. *damselae* biofilm vaccine and evaluated the protective efficacy of oral biofilm vaccines. We confirmed the formation of biofilm on chitosan particles using SEM.

Oral administration is the simplest and most easily applied vaccination method for aquatic animal farmers. Oral vaccines have also been shown to induce antibody responses in salmon [36]. The oral administration of vaccines and adjuvants (e.g., oil-based and aluminum-based adjuvants) has been reported to induce mucosal immunity, particularly in the gut, and to achieve a good protective effect [37,38,39,40]. One report points out that the application of oral biofilm vaccines on different fish is associated with a strong protective effect [12].

Phagocytosis, lysozyme activity, and the A/G ratio are important immune responses to evaluate after vaccination in fish. Our results show that compared with the control group, the biofilm vaccine group demonstrated increased phagocytosis by 264%. Another study on goldfish showed that injection of an *Aeromonas hydrophila* biofilm vaccine can increase the phagocytic ability of macrophages [24]. Additionally, chitosan feeding can increase the macrophage phagocytic ability in sea bass [41]. Serum proteins are divided into two groups: albumin and globulins. The gamma globulin fraction is the source of almost all the serum biochemical active protein. Globulins, such as gamma globulins, are essential for maintaining a healthy immune system [42]. Increases in albumin and globulin levels are considered indicators of a strong innate response in fish [43]. The A/G ratio findings showed that globulin levels increased significantly after treatment with chitosan particle, whole-cell, and biofilm vaccines. Therefore, we believe that increased globulin levels are associated with antibody production. At 28 days after vaccination, the lysozyme levels of the PWV group increased, but there was no significant difference among the groups. In a study of the *Vibrio hollisae* oral vaccine, it was found that an oral whole-cell vaccine could enhance lysozyme activity [44].

Antibody production is an important immune response in fish and can enable fish to resist infection by pathogenic bacteria. Previous research on oral vaccines has shown that when antigens are delivered via the gut, local and systemic immune responses will be elicited, which is reflected in high amounts of circulating IgM [14]. In our study, the biofilm vaccine was associated with a significant increase in IgM at 28 days after vaccination. However, there was no specific antibody production in the other groups. We conclude that the biofilm vaccine stimulates antibody production from GALT. Other studies have shown that *Vibrio anguillarum*, *Aeromonas hydrophila*, and *Streptococcus agalactiae* biofilm vaccines can increase host antibody production [16,18,45,46].

TLRs play crucial roles in the innate immune system by recognizing pathogen-associated molecular patterns derived from various microbes [47]. In humans, TLR3 can only recognize viruses, while in fish, TLR3 not only responds to viral invasion but it also recognizes bacterial pathogen-associated molecular patterns (PAMPs) [48]. As demonstrated by our study, oral PBV is associated with upregulated TLR3 mRNA expression, which in turn increases the downstream IL-1 gene expression [49]. There was no significant intergroup difference in MHC-II expression. Moreover, we found that oral biofilm vaccination can induce IL-8 expression. The trend of CP in TLR 3 and TNF-α is similar to that of PBV; therefore, CP is a good adjuvant that can effectively help PBV to stimulate immune factor responses associated with biofilm vaccine administration that can induce various immune responses.

Finally, the challenge experiment showed that 20 days after challenge, the RPS of the biofilm vaccine group reached 62%. The results of our experiments also observed that no significant antibody production was observed in the PWV after 28 days of vaccination. Therefore, we believe that PWV cannot have a protective effect for a long time. In addition, we also found that on the third day after the challenge, we observed that the fish in the PWV and PBS groups had clinical symptoms such as dark body color and loss of balance, while deaths began to appear on the fifth day, so we think it is a phenomenon caused by individual differences that does not affect the final survival rate result. Although the trend of CP and PBV at the beginning is very similar, in the peak period of 10–15 days after challenge, CP lacks the protection of specific antibodies, so it cannot play a long-term protective effect in the follow-up. At present, most of the reports on oral biofilm vaccines concern *Aeromonas hydrophila* and all have good protective effects [45,46]. To our knowledge, before the present study there were no published studies on vaccines against *Photobacterium damselae* subsp. *damselae* in giant grouper. We showed that oral biofilm vaccines have the potential to be applied to aquaculture.

## 5. Conclusions

We developed a new and effective oral PBV. In our previous work, we confirmed the protective effect of the *Lactococcus garvieae* biofilm vaccine. This time, we tried to apply this method to a common aquatic pathogen. The oral PBV significantly increased specific IgM titers, enhanced phagocytosis, and induced proinflammatory gene expression. This research provides a new method for further development of vaccines that is convenient and suitable for the aquaculture industry.

## Figures and Tables

**Table 1:** Primer reference.

Name	Sequence	Reference
IL-1β-F	CGACATGGTGCGGTTTCTCT	[27]
IL-1β-R	CTCTGCTGTGCTGATTACCAGTT	
TNF-α-F	GCTGCGGCTCGAAGACAAT	[27]
TNF-α-R	CAGACGGTGCGGATGGAGT	
TLR 3-F	TCTCCATTCCGTCACCTTCC	[26]
TLR 3-R	TCATCCAGCCCGTTACTATCC	
MHC-II-F	CCACCCGAACAAACAGACC	[26]
MHC-II-R	TGATGCCCCCTCCAACACT	
IL-8-F	AGTCATTGTCATCTCCATTGCG	[28]
IL-8-R	AAACTTCTTGGCCTGTCCTTTT	
β-actin-F	TACGAGCTGCCTGACGGACA	[26]
β-actin-R	GGCTGTGATCTCCTTCTGC	

**Table 2:** The final mortality and relative percent survival (RPS) value in vaccinated fish.

Group		Final Mortality (%)	RPS (%)
PBV	*n* = 25	32	62
PWV	*n* = 25	84	0
CP	*n* = 25	84	0
PBS	*n* = 25	84	0

## Data Availability

The data for this study are available in this article.
